# Total exudative retinal detachment in a child with pars planitis- a challenging case with optimistic results

**DOI:** 10.1186/s12348-023-00328-3

**Published:** 2023-02-27

**Authors:** Ana Navarrete, Tareq Jaouni, Radgonde Amer

**Affiliations:** Department of Ophthalmology, Hadassah Medical Center, Jerusalem, Israel

**Keywords:** Pars Planitis, Exudative Retinal Detachment, Uveitis, Buckle, Immunosuppressants

## Abstract

We describe a case report of pediatric pars planitis complicated with massive exudative retinal detachment (ERD). A 7-year-old presented with visual acuity (VA) in the right eye (RE) of 6/9 and in the left eye (LE) 6/15. Fundoscopy revealed BE inferior retinoschisis, vitritis and snowballs. He was treated with systemic immunosuppressants. RE retinoschisis resolved within 2 months. Three years later presented with LE VA 6/60 and total ERD. Systemic and intravitreal steroids were administered. Due to refractoriness, he underwent 360° scleral buckle and drainage of subretinal fluid. No retinal breaks or traction were detected. Five months postoperatively LE VA was 6/7.5. Long-term stable outcome was maintained. We report a challenging total ERD as a complication of pars planitis. Although extensive and non-responsive to medical therapy, complete resolution and improvement in vision was achieved with surgical intervention and systemic immunosuppression. We speculate that uncontrolled chronic vasculitic process culminated in diffuse ERD.

## Introduction

Retinal detachment (RD) is an uncommon complication of pars planitis (PP), reported in up to 10% of the cases [1–3]. PP is characterized by chronic inflammation in the pars plana. Inflammation and peripheral retinal ischemia induce angiogenesis and neovascularization. The chronic leakage from the telangiectatic retinal vessels results in intraretinal edema, which leads to the accumulation of subretinal fluid and the development of exudative retinal detachment (ERD).^ 4^

We aim to describe a rare presentation of PP in the form of total ERD, refractory to medical therapy thus necessitating surgical intervention with complete resolution.

## Case report

A 7-year-old healthy boy was referred to the uveitis clinic because of bilateral uveitis discovered on a routine eye exam. On presentation, he was treated with prednisone (25 mg/day). On examination, visual acuity (VA) in the right eye (RE) was 6/9 and in the left eye (LE) was 6/15. Near vision was RE J1 + and LE J7. Anterior segments were normal. Fundoscopy revealed bilateral vitritis, snowballs and bilateral inferior retinoschisis. Spectral-domain optical coherence tomography (SD-OCT) showed normal foveal contour bilaterally. There was no subjective or objective evidence of an underlying disease. Systemic work-up including complete blood count, erythrocyte sedimentation rate, C-reactive protein, Angiotensin-converting enzyme, liver, and kidney function tests, chest x-ray, and serology for Syphilis, Toxoplasma and Toxocara was un-yielding.

**Fig. 1:**
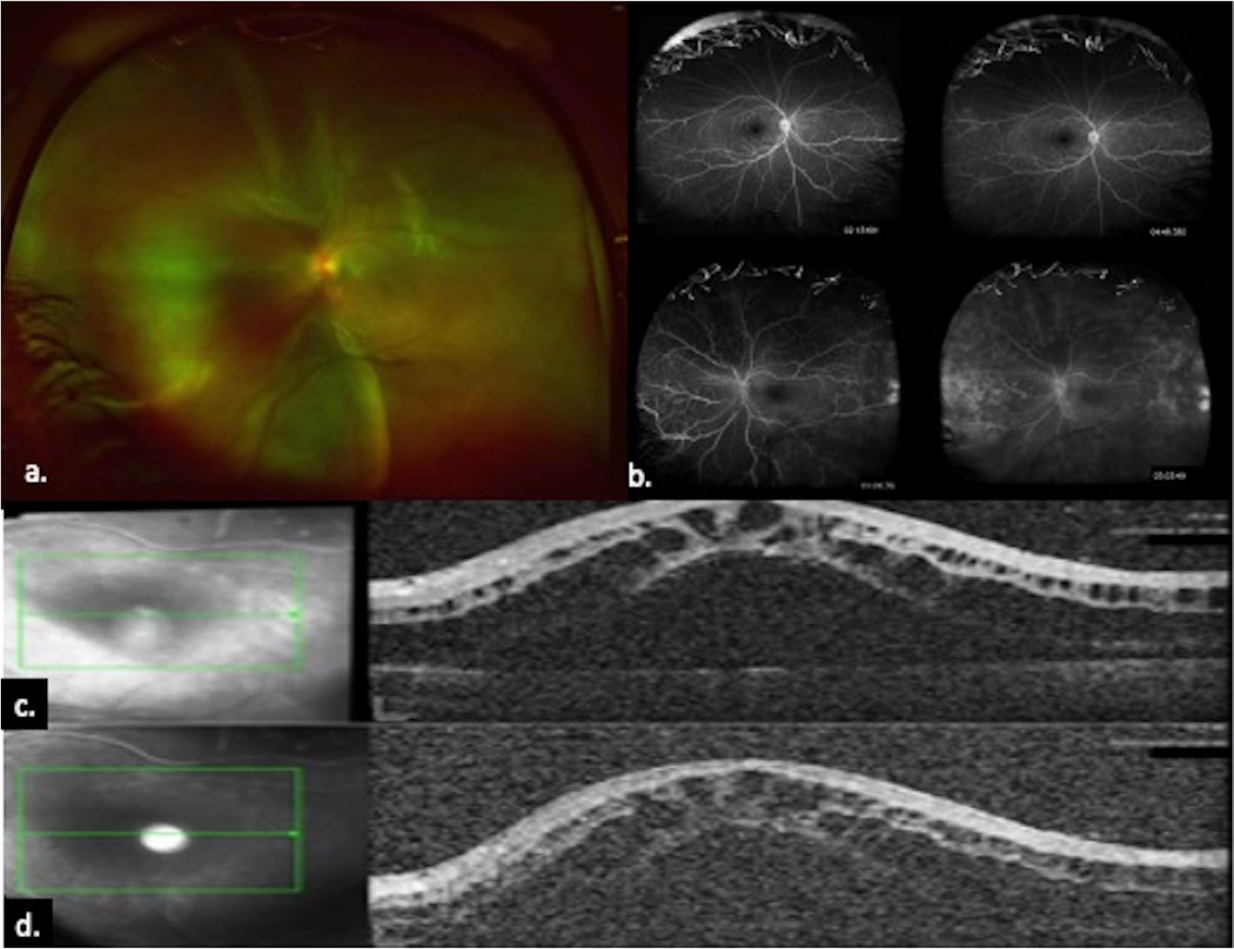
**a** LE Fundus picture shows total exudative retinal detachment, **b** Fluoresceine Angiography shows profuse vascular leakage in the LE, **c** OCT at presentation shows macular detachment with intra- and subretinal fluid, **d** OCT shows lack of improvement after the institution of intravenous steroids, methotrexate and intravitreal triesence

**Fig. 2:**
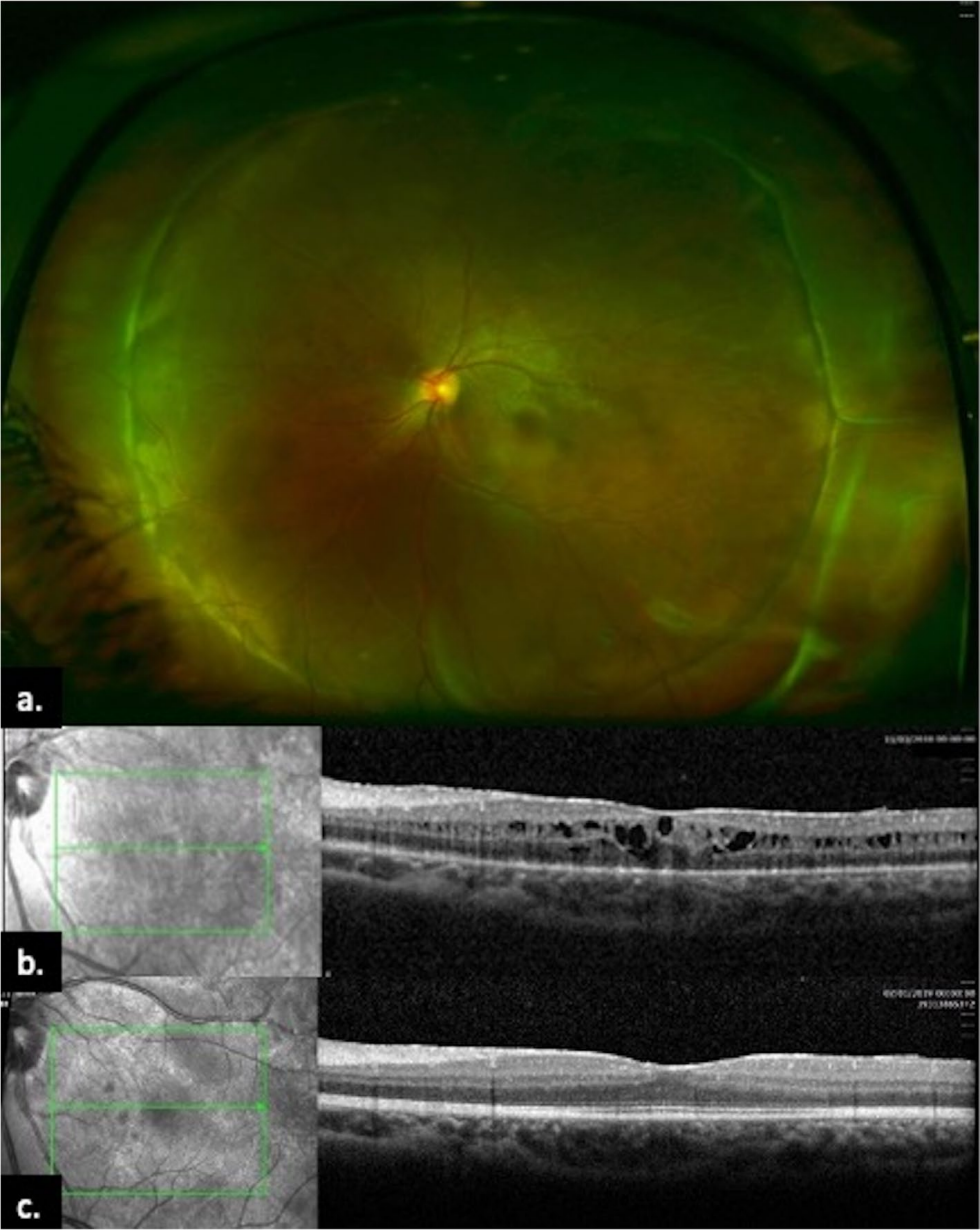
**a** One year After surgical intervention flat retina with good 360° scleral indentation, **b** three weeks after surgery shows marked resolution of intra- and subretinal fluid and resolution of macular detachment, **c** two years after surgery shows normal looking foveal contour

The child was diagnosed as having PP-associated inferior retinoschisis. Oral methotrexate was introduced as a steroid-sparing agent and prednisone tapered. Two-months later, the RE retinoschisis resolved. Five-months later, RE VA was 6/7.5 and LE VA was 6/12 and near vision was J1 in each eye. Subsequently, the child was lost to follow-up and presented 3 years later with LE drop in vision of 3 week-duration. During these years the parents of the patient discontinued methotrexate. On examination, RE VA was 6/7.5, LE VA was 6/60. Near vision was RE J1, LE J16. RE fundoscopy revealed snowballs inferiorly. LE biomicroscopy showed active anterior-uveitis. LE fundoscopy revealed total RD (Fig. 1a). No retinal breaks or tractional membranes were seen and no choroidal-detachment. Fluorescein angiography (FA) (Fig. 1b) revealed profuse vascular leakage all over the left fundus with intense leakage in the nasal and temporal periphery. SD-OCT (Fig. 1c) showed left macular detachment with intra and subretinal fluid. The child was diagnosed with total ERD. Treatment was initiated with a three-day pulse of intravenous methylprednisolone (500 mg/day). Methotrexate was instituted and prednisone was introduced subsequently. Two-weeks later because of lack of clinical improvement, intravitreal triamcinolone acetonide was injected (4 mg/0.1 cc). Because of the persistence of total ERD and refractoriness to medical therapy (Fig. 1d), he underwent surgical repair with 360° encircling scleral buckle and drainage of subretinal fluid. No vitrectomy or tamponade was needed. No retinal breaks or tractional membranes were detected intraoperatively. One-month postoperatively, LE VA was 6/15. LE fundoscopy showed flat retina and marked resolution of intra and subretinal fluid (Fig. 2a and 2b) Five months postoperatively LE VA was 6/7.5 and J1 for near. Two years postoperatively, the LE remained with VA 6/7.5 and J1. There was no recurrence of ERD and Methotrexate was continued as a monotherapy (Fig. 2c).

## Discussion

We report a rare complication of pediatric PP in the form of total ERD developing 3.5-years after the 1^st^ presentation. It was extensive, long-standing, and unresponsive to intensive systemic and local steroid therapy. Surgical intervention was subsequently required with complete resolution of ERD. Stable visual and surgical outcomes were maintained over a two-year follow-up period. We speculate that diffuse uncontrolled chronic vasculitic process was the underlying mechanism that culminated in total ERD.

We have recently published a cohort of 33 children (58 eyes) with pars-planitis in whom ERD occurred in 7% of the eyes [2]. None of them had ERD at presentation but developed it during the follow-up time. Pollack et al., reported on 13 eyes with pars planitis-associated peripheral retinal elevation [4]. They described it as a consequence of long-standing inflammation. Brockhurst [5] described that retinoschisis in chronic peripheral uveitis developed after several years of active disease, with massive exudation overlying the inferior portions of the ora serrata. Treatment of this form of retinoschisis was rarely necessary, with only one case needing wide scleral buckling with subsequent drainage of fluid due to progression [5]. Similarly, Pollack et al. [4] reported that the schisis/ERD remained stable in 9 of 13 eyes. The authors theorized that peripheral retinal elevation was the result of vascular abnormalities with intraretinal fluid leaking from telangiectatic retinal vessels.

In addition to the exudative mechanisms of peripheral retinal elevations, tractional mechanisms were previously described. It is hypothesized that traction occurs due to contraction of snowbanking caused both tangential and radial traction on the pars plana and peripheral retina leading to peripheral retinal elevation. The elevation subsequently relieves further traction, explaining the lack of progression in the majority of cases [5, 6].

Interestingly, PP-associated rhegmatogenous RD (RDD) has been shown to have a large number of retinal breaks, mainly composed of round holes, and a greater extent compared to primary RRD cases [7]. Although one study showed that the primary anatomic success rate was lower in PP associated RRD than primary RRD (78.1% versus 92.7%), the final anatomic success rate was comparable (96.8% and 97.7% respectively). However, PP associated RRD group showed a higher rate of postoperative proliferative vitreoretinopathy compared to the primary RRD group [7].

In children, PP is often asymptomatic and on the first encounter with the ophthalmologist, they are discovered to have a plethora of anterior and posterior segment complications. Systemic immunosuppressive treatment and accurate follow-up can prevent the development of vision-threatening complications. It is highly possible that the child in the present report had long-standing inflammation that culminated in bilateral inferior retinoschisis at the time of 1^st^ presentation to the uveitis clinic. FA performed at the time of total ERD demonstrated an extensive inflammation of the retinal vasculature, which was marked in the periphery of the retina. No vitreous traction or retinal breaks were observed at the time of surgical repair and visual outcome after the surgery was favorable over a two-year follow-up period.

## Data Availability

Not applicable.

## References

[1] Malinowski SM, Pulido JS, Folk JC (1993). Longterm visual outcome and complications associated with pars planitis. Ophthalmology.

[2] Navarrete A, Koriat A, Amer R (2020). Implications of pars planitis-associated cystoid macular edema on visual outcome and management in children. Graefes Arch Clin Exp Ophthalmol.

[3] Ozdal P, Tugal-Tutkun BN, I,  (2015). Pars planitis: epidemiology, clinical characteristics, management and visual prognosis. J Ophthalmic Vis Res.

[4] Pollack AL, McDonald HR, Johnson RN (2002). Peripheral retinoschisis and exudative retinal detachment in pars planitis. Retina.

[5] Malalis JF, Bhat P, Shapiro M, Goldstein DA (2016). Retinoschisis in pars planitis. Ocul Immunol Inflamm.

[6] Jalil A, Dhawahir-Scala FE, Jones NP (2010). Nonprogressive tractional inferior retinal elevation in intermediate uveitis. Ocul Immunol Inflamm.

[7] Kim YK, Yoon W, Ahn JK, Park SP (2016). Scleral Buckling for Rhegmatogenous Retinal Detachment Associated with Pars Planitis. J Ophthalmol.

